# International Survey on Phenylketonuria Newborn Screening

**DOI:** 10.3390/ijns11010018

**Published:** 2025-02-26

**Authors:** Domen Trampuž, Peter C. J. I. Schielen, Rolf H. Zetterström, Maurizio Scarpa, François Feillet, Viktor Kožich, Trine Tangeraas, Ana Drole Torkar, Matej Mlinarič, Daša Perko, Žiga Iztok Remec, Barbka Repič Lampret, Tadej Battelino, ISNS Study Group on PKU, Francjan J. van Spronsen, James R. Bonham, Urh Grošelj

**Affiliations:** 1 Clinical Institute for Special Laboratory Diagnostics, University Children’s Hospital, Ljubljana University Medical Center, Vrazov trg 1, 1000 Ljubljana, Slovenia; 2 International Society for Neonatal Screening, Reigerskamp 273, 3607 HP Stichtse Vecht, The Netherlands; 3 Center for Inherited Metabolic Diseases, Karolinska University Hospital, SE-171 76 Stockholm, Sweden; 4 Department of Molecular Medicine and Surgery, Karolinska Institutet, SE-171 76 Stockholm, Sweden; 5 Regional Coordinator Centre for Rare Diseases, University Hospital of Udine, 33100 Udine, Italy; 6 Pediatric Unit, Reference Center for Inborn Errors of Metabolism, University Hospital of Nancy, 54500 Nancy, France; 7 INSERM UMRS 1256, Nutrition, Genetics, and Environmental Risk Exposure (NGERE), Faculty of Medicine of Nancy, University of Lorraine, 54505 Nancy, France; 8 Department of Pediatrics and Inherited Metabolic Disorders, Charles University-First Faculty of Medicine and General University Hospital in Prague, Ke Karlovu 2, 128 08 Praha 2, Czech Republic; 9 Norwegian National Unit for Newborn Screening, Division of Pediatric and Adolescent Medicine, Oslo University Hospital, 0424 Oslo, Norway; 10 Department of Endocrinology, Diabetes and Metabolic Diseases, University Children’s Hospital, Ljubljana University Medical Center, Bohoriceva 20, 1000 Ljubljana, Slovenia; 11 Faculty of Medicine, University of Ljubljana, Vrazov trg 2, 1000 Ljubljana, Slovenia; 12 Division of Metabolic Diseases, Beatrix Children’s Hospital, University Medical Center Groningen, University of Groningen, 9718 GZ Groningen, The Netherlands; 13 Sheffield Children’s (NHS) FT, Sheffield S10 2TH, UK; 14 Center for Rare Diseases, University Children’s Hospital, Ljubljana University Medical Center, Bohoričeva 20, 1000 Ljubljana, Slovenia

**Keywords:** phenylketonuria, newborn, neonatal, screening, international, survey, laboratory, methods, cut-off

## Abstract

Newborn screening for Phenylketonuria enables early detection and timely treatment with a phenylalanine-restricted diet to prevent severe neurological impairment. Although effective and in use for 60 years, screening, diagnostic, and treatment practices still vary widely across countries and centers. To evaluate the Phenylketonuria newborn screening practices internationally, we designed a survey with questions focusing on the laboratory aspect of the screening system. We analyzed 24 completed surveys from 23 countries. Most participants used the same sampling age range of 48–72 h; they used tandem mass spectrometry and commercial non-derivatized kits to measure phenylalanine (Phe), and had non-negative cut-off values (COV) set mostly at 120 µmol/L of Phe. Participants mostly used genetic analysis of blood and detailed amino acid analysis from blood plasma as their confirmatory methods and set the COV for the initiation of dietary therapy at 360 µmol/L of Phe. There were striking differences in practice as well. While most participants reported a 48–72 h range for age at sampling, that range was overall quite diverse Screening COV varied as well. Additional screening parameters, e.g., the phenylalanine/tyrosine ratio were used by some participants to determine the screening result. Some participants included testing for tetrahydrobiopterin deficiency, or galactosemia in their diagnostic process. Results together showed that there is room to select a best practice from the many practices applied. Such a best practice of PKU-NBS parameters and post-screening parameters could then serve as a generally applicable guideline.

## 1. Introduction

Phenylketonuria (PKU; OMIM 261600) is an inborn error of metabolism caused by mutations in the phenylalanine hydroxylase (*PAH*) gene. In healthy individuals, PAH converts phenylalanine (Phe) into tyrosine (Tyr) requiring tetrahydrobiopterin (BH4) as a co-factor in the reaction [1,2]. Defects in the metabolism of Phe result in elevated concentrations of this amino acid in the blood of affected patients, a condition known as hyperphenylalaninemia (HPA), which is also the underlying cause of neurophysiological defects [3]. PKU is treated through dietary therapy, which consists of restriction of natural protein intake, supplementation with a Phe-free amino acid mixture, and consumption of low-protein food [4]. Early detection and dietary treatment are crucial to prevent severe symptoms, decrease morbidity and mortality, and dramatically improve patients’ quality of life [1,2,4]. Early detection of HPA in the population of newborns can be achieved through ubiquitous screening programs [1,2]. More than 60 years ago Robert Guthrie enabled widespread newborn screening for hyperphenylalaninemia (HPA), by adapting a bacterial growth inhibition test to detect elevated concentrations of Phe in dried blood spot (DBS) samples of newborns [1,2,4]. Since then several other analytical methods to measure Phe concentrations were developed, including tandem mass spectrometry (TMS), fluorimetric, radioimmuno assays, and enzymatic tests [1,2,5,6]. In over 60 years, apart from a variety of laboratory tests, a variety in other parts of the PKU newborn screening process (PKU-NBS), e.g., diagnostic follow-up and treatment, inevitably ensued, allowed by a lack of international guidelines [1,2,7,8,9,10,11,12]. To assess the state of PKU-NBS approaches in Europe and beyond, we conducted an online survey which focused mainly on the laboratory aspect of the screening system (Document S1: Survey questionnaire). We additionally inquired about the way that centers differentiate HPA and confirm PKU and how they define a true positive. The survey was sent to a selection of members of the International Society for Newborn Screening (ISNS), a global society whose members are professionals in the field of newborn screening (https://www.isns-neoscreening.org, accessed on 26 November 2024). The aim of the survey and this study was to reveal the similarities and differences in PKU-NBS practice. Results together were used to determine the most common practices, allowing for establishing guidelines for acceptable and best practices in PKU-NBS. With such guidelines, the quality of PKU-NBS can be improved globally.

## 2. Materials and Methods

### 2.1. Creation and Distribution of the Survey

We designed a survey focusing mostly on the following parts of the screening system; the laboratory test used for screening, cut-off values (COV) for positive screening results, and laboratory diagnostic procedures (distinguishing between HPA and PKU). The survey was reviewed by several members of ISNS and was amended according to their suggestions, it was then reviewed once again by the same reviewers before it was published online on the RedCap^®^ platform (https://project-redcap.org, accessed on 11 May 2023). The survey was then distributed to members of ISNS, representing newborn screening (NBS) centers from different countries and institutions in 51 countries geographically located in Europe and Central Asia. Participants were encouraged to provide or ask for additional clarifications regarding either questions or answers. The initial submission deadline was extended to give participants who were previously unable to submit an opportunity to respond.

### 2.2. Data Cleanup, Unification, and Analysis

Only fully completed surveys were included in the analysis. Some participants requested amendments to their answers via e-mail, which were included. In case clarifications were needed, participants were contacted through e-mail as well. Free-form answers were curated for the purposes of analysis. Examples of such cases are as follows: translating units mg/dL to µmol/L Equation (1), unifying descriptions of methods, and answers submitted under the option “Other”. We consolidated single or multiple-choice answers with free-form answers where applicable. An example of such consolidation is the use of confirmatory methods, where some centers conduct tests within the center while others outsource them. In this analysis, both scenarios were considered as the center utilizing the test, regardless of whether it was performed on-site or outsourced. Answers were analyzed using Microsoft Excel 2016 64-bit edition, and graphs were drawn using RStudio v. 2024.04.2 Build 764 [13], based on R v. 4.4.1 [14]. Packages used were tidyr [15], ggplot2 [16], reshape [17], gridExtra [18], and dplyr [19].

(1) $$Concentration\left(\frac{µmol}{L}\right)=\left(\frac{Concentration(mg/dL)}{Phe\;Molecular\;Weight\;(165.19\;g/mol)}\right)$$

### 2.3. Ethical Statement

The study did not involve any clinical research or the use of participants’ personal or medical information. All survey information was provided voluntarily, with participants giving their consent for the results to be published.

### 2.4. Definition of Terms

Hyperphenylalaninemiaor HPA is a medical condition where the concentration of Phe in blood is elevated above the COV set by the NBS center.Phenylketonuriaor PKU is a diagnosis when HPA fits the definition of a true positive case of PKU as defined by the center/hospital treating the newborn.Cut-off value or COV are values that define at which concentration of Phe a screening result is positive, i.e., a newborn is suspected to have HPA. Some participants use a single value for COV, while others use a range where the concentration of Phe is regarded as elevated but not yet necessarily a marker of actual HPA.Sampling ageis the range of ages (in hours) at which a sample is obtained. Blood samples acquired outside this range are usually deemed not fit for analysis.Confirmatory methodsmethods which are used to confirm HPA; differential tests enable the analysis of the source of HPA.The Phe/Tyr ratioa parameter that enables the elimination of non-physiological fluctuations of Phe or Tyr and differences in blood volume between DBS, as well as inadequate elution of analytes from DBS [20], increasing testing sensitivity and specificity [21]. This is due to the biochemical pathway of Phe metabolism, as Tyr concentrations in blood reflect PAH function [1,2,8,22] and is therefore a marker of its activity.Borderline resultan analytical result that is beyond the value set for a negative screening result and below the value set for a positive result. It is treated as less severe and typically warrants a different response than a positive result would have.

## 3. Results

### 3.1. Response Rate

After reviewing complete responses and including amendments to the answers, a total of 26 surveys were answered in full, yielding a response rate of approx. 51%. The countries represented were Austria, Belgium, Croatia, Cyprus, Estonia, Finland, France, Georgia, Hungary, Iceland, Kazakhstan, Lithuania, Luxembourg, Malta, Netherlands, Norway, Poland, Romania, Slovak Republic, Slovenia, Spain, Sweden, and Uzbekistan, with France contributing two answered surveys from two different centers. Two of the surveys were not included in the analysis, one survey was submitted by Kosovo but has not been included in statistical analysis as they did not run a PKU-NBS program and one of the participants declined inclusion in the article. We initially received two responses from France (two centers responded to the survey); however, they were later asked to consolidate the response as their screening guidelines were the same throughout the country. The final number of participants in the statistical analysis was 23.

### 3.2. Sampling Age

**Figure 1:**
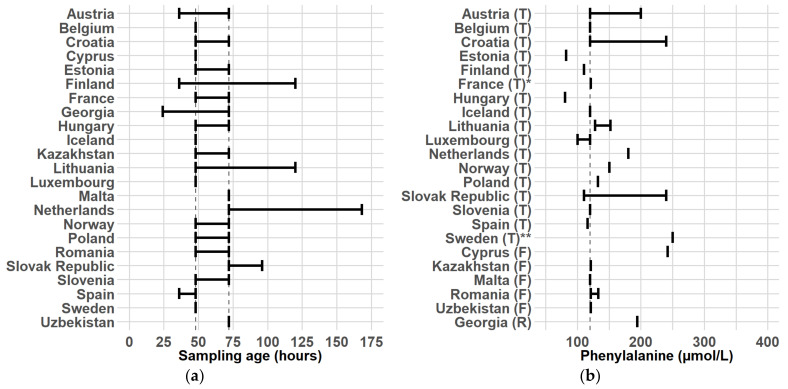
(**a**) Sampling age ranges of participating countries/centers. The vertical dashed lines represent the range 48–72 h; (**b**) Phe COVs, at which the screening result is no longer negative, by the method. The vertical dashed line represents the value 120 µmol/L. Participants with ranges utilized a borderline result, where the range produced a borderline result. T–TMS; F–Fluorometric; R–Radioimmuno assay * France planned to update their COV by the end of 2024 to 150 µmol/L. ** Sweden used another criterion: if both Phe is above 180 µmol/L and Phe/Tyr ratio is above 2, the screening result is positive.

The earliest reported sampling age was 24 h (h) and the latest was 168 h with the most commonly reported range being 48–72 h (n = 9; 39%) Figure 1a. A single time point was reported by seven participants as their sampling age, with five of them reporting a sampling age of 48 h and two reporting 72 h.

### 3.3. Laboratory Methods

TMS was used by 17 participants (74%). Other methods used were the fluorometric method (n = 5; 22%) and radioimmunoassay (n = 1; 4%). Reagent kits used varied mostly when using TMS systems. Most participants (n = 9; 53%) used commercial non-derivatized kits for TMS, three (18%) used commercial derivatized kits, and four (23%) used in-house developed derivatized kits.

### 3.4. COV, Updates, Borderline or Positive Result

The range of reported cut-off values (COV) was 80.46 µmol/L–250 µmol/L. The median value at which the result was no longer considered negative was 120 µmol/L. Most reported COVs (n = 14; 58%) fell within the range of 110–130 µmol/L (n = 14; 58%). A minority of participants (n = 6; 26%) applied a category ‘borderline results’ Figure 1b. Most participants (n = 11; 48%) had not updated their COV. Occasional updates were performed by eight (35%), and regular updates by four (17%) participants, three of those updated their COV every year and one every half year. Of those who occasionally update, seven provided a reason. Of these seven, three participants responded that the change was a regular update, three because of method change, and one because of a high false-positive rate.

### 3.5. Confirmatory Methods

**Figure 2:**
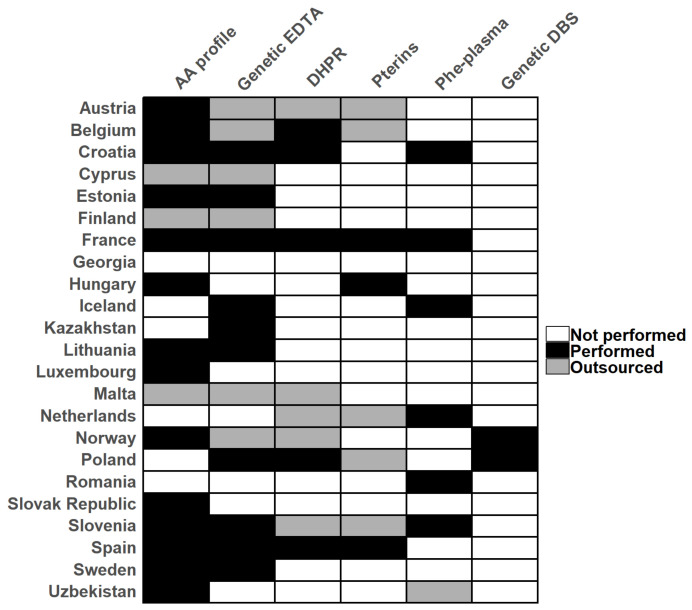
Confirmatory methods used by centers. Genetic EDTA: genetic testing from EDTA blood; AA profile: detailed amino acid profile analysis; Pterins: biopterin and neopterin measurement/analysis from urine; Phe-plasma: Phe from blood plasma; Genetic DBS: genetic testing from DBS.

Combining multiple-choice and free-form answers, 17 (74%) conduct detailed amino acid analysis (amino acid profile) from blood plasma, 16 (70%) participants utilize genetic analysis from EDTA blood, and 7 (30%) measure Phe and Tyr from blood plasma Figure 2. Measuring dihydrobiopterin reductase (DHPR) activity in red blood cells from DBS was utilized by ten (43%) participants, while eight (35%) participants measured neopterin and biopterin after the BH4 loading test, and one measured before the loading test. Additionally, two (8%) utilized genetic analysis from DBS.

### 3.6. Phe/Tyr Ratio in PKU-NBS

A total of 15 (65%) participants used the Phe/Tyr ratio, seven (30%) did not, and one (4%) only in some cases. The range of COV reported was 1.0 to 2.37 (median:1.50). Participants who occasionally used the ratio do so when considering a positive screening result if the ratio was above 2, and Phe was above 180 µmol/L.

### 3.7. Testing for Galactosemia After Positive PKU-NBS

To exclude elevated Phe due to galactosemia, only seven (30%) participants were tested for this disease in some capacity. Of these, three (13%) test always and four (17%) test occasionally (two when clinical presentation warrants it and one in case of increased methionine and Tyr). Most participants (n = 16; 69%) however, do not test for galactosemia due to elevated Phe. This result could, however, be influenced by the way the question was posed, as we specifically asked if participants were tested for galactosemia due to elevated Phe and not if they performed NBS for galactosemia.

### 3.8. Testing for BH4 Deficiency upon Exceeded Phe COV

Sixteen participants (70%) were tested for BH4 deficiency in some capacity, with nine (39%) participants were tested in all cases and seven (30%) in some cases. Of those seven, two participants test when pathological variants are not found in PAH alleles, with one testing when only one or no pathogenic variants of the PAH gene are found, if Phe concentration in blood are above 150 µmol/L, or if no other genes that may cause increased Phe are found to be mutated. Another participant was tested if Phe was over 600 µmol/L in case the pterine profile was delayed. One center only performs the test if it is paid for by the parents or caregivers. Seven participants (30%) do not test for BH4 deficiency.

### 3.9. Additional Diagnostic Resources

Nine participants (39%) used various additional means to help in decision-making between screening and diagnosis of which seven (30%) used CLIR (Collaborative Laboratory Integrated Reports, by Mayo Clinics, Rochester (MN), https://clir.mayo.edu, accessed on 18 November 2024) to aid in diagnosis if needed and one (4%) participant used Orphanet (https://www.orpha.net, accessed on 18 November 2024) and BioPku (http://www.biopku.org, accessed on 18 November 2024). Others did not state their resources. Fourteen (61%) of participants did not use such resources in diagnostics.

### 3.10. Definition of a True Positive Case of PKU

Nine (39%) participants defined a true positive case of PKU as a patient with pathological variants in both *PAH* alleles. Seven (30%) defined a true positive as a patient with Phe blood concentrations persistently above the COV for HPA, and four (17%) defined a true positive as a patient needing dietary therapy. One participant defined true positivity as a need for dietary therapy and the presence of pathological variants in both *PAH* alleles. One participant based the true positive diagnosis on persistently elevated Phe concentration and pathological variants in *PAH* alleles; however, they also considered BH4 deficiency as a true positive case.

### 3.11. COV for Introducing Dietary Therapy

**Figure 3:**
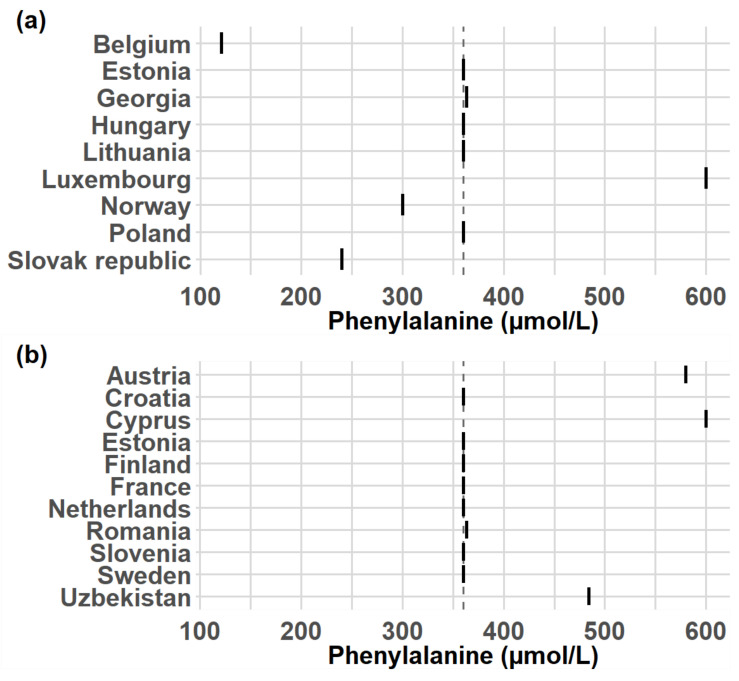
Phe COVs, at which therapy is indicated. (**a**) COVs of centers that measure Phe in DBS. (**b**) COV of centers that measure Phe in blood plasma. This information is used to help the designated physician determine whether the therapy should be initiated.

The median COV above which the start of dietary therapy was indicated was 360 µmol/L, with 11 (58%) of participants setting COV either at 360 µmol/L or 363 µmol/L (6 mg/mL. n = 2; 9%). The range of reported COV was 121 µmol/L (approx. 2 mg/dL)–600 µmol/L (approx. 9.9 mg/dL). Among the participants who utilized this parameter, ten (43%) of them measured Phe using DBS, and 11 (48%) measured it in plasma Figure 3. Two participants did not provide a COV and one indicated that the decision was made by the physician.

## 4. Discussion

In this study, we aimed to assess the similarities and differences in PKU-NBS and diagnostic practices, focusing on the laboratory aspect of the process.

The results of the survey show that the most common time frame for sample collection is 48–72 h after birth, which is in accordance with previously suggested guidelines [2,4]. Nevertheless, varying sample time ranges were reported, some starting earlier than 48 h or allowing sampling as late as 168 h. With samples taken too early, HPA might go unnoticed as prior to 48 h the newborn is not at its normal metabolic capacity and thus Phe blood concentrations can be lower and not represent stabilized physiological levels [23]. Notably, it is advised that dietary treatment of cases should start before the age of 10 days [4]; therefore, very late sampling should also be avoided to allow for timely confirmatory testing and differential diagnostics.

Among the laboratory methods used to measure Phe concentration, TMS was preferred. TMS offers significant advantages over other methods by enabling the measurement of numerous analytes simultaneously [24,25], allowing for the screening of multiple metabolic diseases at once, and providing superior analytical sensitivity. Fluorometric methods were the second most used. These methods allow measuring of only a single analyte, in this case, Phe; therefore, if the measurement of another analyte is needed, a separate kit has to be used [23]. Differences in COVs can be explained by the various methodologies used to measure Phe [5,26,27,28]. It is therefore important for laboratories to be involved in interlaboratory comparison programs and share their results with other laboratories.

COVs are a key component of laboratory screening procedures. Our study observed that a COV of 120 µmol/L (or approx. 2 mg/dL), which has previously been suggested as the definition of HPA [1,2,4], is used by the majority of participants either as COV for a positive screening result or the lower value of the borderline range. Despite this, we observed a broad range of COVs, which can prove problematic. Low COV is often chosen as an additional cautionary measure to not miss cases where Phe is not yet sufficiently increased. Low COV, however, poses a risk of unnecessary recalls, confirmatory testing, and over-hospitalization, presenting additional costs to the system [26] and more importantly, causing long-lasting stress to the parents by a perceived high vulnerability of the child’s health [29]. A particularly high COV can, in contrast, increase the risk of false negatives [5,26,30], leading to cases of PKU being overlooked. Although the highest reported COV for a positive screening result was below the therapeutic threshold of 360 µmol/L [1,2,4,8], this can still result in a false-negative result [30].

Many participants occasionally or regularly updated their COVs due to high false-positive rates, changes in methodology, or as part of regular updates. In our study, most participants who initially had very high or low COVs had already updated them.

A minority of participants applied, as a cautionary measure, the category of ‘borderline results’ (next to COVs) in their screening algorithm. Most commonly, with a borderline result, participants repeated the analysis from the initial sample or from a new sample prior to performing any confirmatory tests or inviting the newborn and parents/caregivers to the clinic or NBS center for further evaluation. This way, the centers spare parents unnecessary stress of a positive result that might be an analytical error or only transiently elevated Phe. Conversely, this additional step does prolong the final diagnosis. Repeating analysis from the initial card should always be performed to confirm that elevated Phe is not a consequence of an artifact, such as measurement errors, errors in sample acquisition, sample preparation, and similar factors.

Another parameter to screen for HPA or PKU is the Phe/Tyr ratio. This ratio is used by the majority of participants. Due to the biochemical pathway of Phe metabolism, Tyr concentrations in the blood reflect PAH function [1,2,8,22,31]. This ratio eliminates non-physiological fluctuations of Phe or Tyr and differences in blood volume between DBS, as well as inadequate elution of analytes from DBS [20], increasing testing sensitivity and specificity [21,31]. The prerequisite for applying this ratio is the availability of either TMS, which measures multiple analytes simultaneously, or a separate test for Tyr on the same analyzer, as is the case with the fluorometric method [21,23,32].

Our survey found that most participants used the Phe/Tyr ratio, with a median COV of 1.51, as suggested in the literature [2,21,32]. However, some ratios were set quite high compared to the median ratio, risking missed HPA or PKU diagnosis if relying solely on the ratio. A robust Phe/Tyr ratio based on broader data could improve the quality of NBS results. The Phe/Tyr ratio is also useful for distinguishing between PKU and persistent HPA [21,32] and detecting PKU in newborns whose samples were taken within 24 h after birth, where HPA or PKU might not yet be detectable through Phe alone [23].

Positive screening results warrant a clinical response. The most common procedures after a positive screening result are obtained are repeating the analysis from the initial or new DBS sample and performing confirmatory and differential analysis in a diagnostic laboratory. In most cases, newborns are invited to the clinic and have a fresh sample taken, from which confirmatory tests are performed. A few centers include second-tier analysis in their procedure, prior to recall. As with borderline results, it is even more important to repeat analysis from the initial sample prior to reporting a positive result in case elevated Phe is a consequence of an artifact. This is especially true if the parents are contacted, as reported positive results could cause stress to the parents [29], as discussed above in the discussion.

Apart from screening, we also inquired about which confirmatory and differential tests participants utilize, regardless of whether they performed the test on-site or outsource to another laboratory. The most commonly used confirmatory method is the amino acid profile analysis in blood plasma. Beyond the advantages of measuring Phe directly in blood plasma, this analysis can determine if the elevated Phe level is due to HPA or PKU, or if it is elevated in combination with other amino acids indicative of other issues in amino acid metabolism [1]. The second most commonly used confirmatory method is genetic testing from EDTA blood. This method can confirm mutations in *PAH* alleles [1,2,25,33]. Moreover, a more thorough genetic analysis can distinguish HPA cases originating from damaged DNAJC12 alleles [2,34]. Caution must however be applied whenever utilizing genetic testing, as it is important to keep the list of mutations up to date [35]. Few participants measured Phe concentration directly from blood plasma, despite its advantages, such as eliminating imprecision from DBS and not requiring a separate reagent kit [8,20].

Among patients with PKU, 1–2% had elevated Phe concentrations due to disorders in the BH4 metabolism, leading to its deficiency [1,2,22,33]. Analysis of DBS or urine for neopterin and biopterin, and measurement of DHPR activity is essential for an accurate diagnosis of this condition [1,2,22,33]. Patients also need to be tested for BH4 responsiveness, as administering BH4 increases the success of treatment [1,2,22,29]. While genetic testing can predict BH4 responsiveness to an extent, a BH4 loading test is a more direct test of responsiveness [1,2,22,33]. Despite a considerable number of participants testing for BH4 deficiency in all or some cases of elevated Phe, a significant number did not, nor did they perform the BH4 loading test. Considering the potential positive impact administering sapropterin (a BH4 supplementation treatment) can have on responders to BH4 treatment, centers would be advised to perform this test [36]

Newborns with elevated Phe concentrations in blood plasma are recommended to be tested for galactosemia if galactosemia is not part of the screening panel [37,38]. Galactosemia in newborns leads to the accumulation of glucose-1-phosphate in the liver, which may lead to liver dysfunction, resulting in elevated concentration of Phe due to the liver’s failure to properly metabolize this amino acid [37]. Testing for galactosemia could therefore help in clarifying if this disease is the underlying factor of elevated Phe concentrations [37,38,39]. The majority of participants stated, however, that they did not test for galactosemia when Phe was elevated. It is, therefore, important to emphasize, that this result could have been influenced by a misunderstanding of the question, where we very specifically asked about performing testing for galactosemia due to elevated Phe as a confirmatory test and not if the center screened for galactosemia in general. This means that a center that performed NBS for galactosemia could have answered negatively to the question, as they performed screening, regardless of Phe levels.

Lastly, a true positive PKU diagnosis is defined by most participants as having pathogenic variants in both *PAH* alleles. To a lesser extent, participants define a true positive as a patient requiring dietary therapy or through a combination of several confirmatory analyses.

A COV is normally used to determine the need for dietary therapy; however, it is ultimately the only information used to guide the designated physician in the decision of whether the diet therapy should be initiated. In our survey, most participants used a COV of 360 µmol/L (approx. 6 mg/dL) for the Phe concentration in blood at which dietary therapy should begin, a suggested in several guidelines [1,2,4,8]. Participants measured this parameter either in DBS or blood plasma. A few participants set the COV for dietary therapy below 360 µmol/L, which can be problematic as it may lead to unnecessary treatment and stress, given the strict and challenging nature of dietary therapy [1,2,40]. Some participants had COVs set above 360 µmol/L, which could prove problematic as well because these values fell within the range of 360–600 µmol/L where the advisability of treating a patient is unclear [1,2,4,8]. However, the differences between these COVs could again be explained by participants using different methodologies to measure Phe [5,8,20].

Participants used different measuring units for Phe concentration both in screening, diagnosis, and treatment; basically, µmol/L and mg/dL were used. For example, in the case of diet therapy, 360 µmol/L was the most commonly used COV, however, a value of 6 mg/dL was also stated, implying that these were to some extent equal. The reason for this is possibly the fact that those are both discrete and/or round numbers, which note the same COV but do not note exactly the same concentration, as 6 mg/dL equals approx. 363 µmol/L, making comparison difficult.

## 5. Conclusions

As we observed, the results of this survey show that, despite many similarities, a high degree of variability is to be found in approaches to the NBS process. This reveals a need for a better exchange of data on COV, sampling age, and other aspects of NBS between different centers. There is also a case to be made for unifying units in which we measure Phe. All these factors reveal a need for accepted official minimal guidelines, which would unify the PKU-NBS process the world over and improve the quality of the screening process.

## Data Availability

Data are available upon request.

## Supplementary Materials

The following supporting information can be downloaded at https://www.mdpi.com/article/10.3390/ijns11010018/s1. Document S1: Survey questionnaire.

## Appendix A

**ISNS Study Group on PKU**: Austria: Austrian Newborn Screening, Department of Pediatrics, Medical University of Vienna (Maximilian Zeyda), Belgium: Centre Hospitalier Universitaire de Liège (Boemer François), Croatia: University Hospital Center Zagreb (Iva Bilandžija Kuš), Cyprus: The Center for Preventive Pediatrics “Americos Argyriou” (Alexia Nicolaou), Estonia: Tartu University Hospital (Karit Reinson), Finland: Screening Centre for Inborn Errors of Metabolism, Turku University Hospital (Riikka Kurkijärvi), France: Hospices Civils de Lyon (David Cheillan) and University Hospital of Nancy (Feillet François), Georgia: MediClubGeorgia (Nazi Tabatadze), Hungary: Semmelweis University (Ildikó Szatmári), Iceland: Landspitali University Hospital (Franzson Leifur), Kazakhstan: Scientific Center for Obstetrics, Gynecology and Perinatology (Gulnara Svyatova), Kosovo: Pediatric Clinic, University Clinical Center Pristina (Vjosa Kotori), Lithuania: Vilnius University Hospital Santaros Clinics (Jurgita Songailiene), Luxembourg: Laboratoire National de Santé (Patricia Borde), Malta: Mater Dei Hospital (Ian Brincat), Norway: Norwegian National Unit for Newborn Screening, Oslo University Hospital (Trine Tangeraas), Poland: Institute of Mother and Child (Mariusz Oltarzewski), Romania: National Institute for Mother and Child Health “Alessandrescu-Rusescu” (Florentina Moldovanu), Slovak Republic: Newborn Screening Center of Slovak Republic, Children hospital Banská Bystrica (Zuzana Mydlová), Slovenia: Ljubljana University Medical Center (Barbka Repič Lampret), Spain: Málaga Regional University Hospital (Raquel Yahyaoui), Sweden: Center for inherited metabolic diseases, Karolinska University Hospital (Rolf Zetterström), The Netherlands: Dutch National Institute for Public Health and the Environment (RIVM) (Rose Maase), Uzbekistan: Republican Center “Mother and child screening” (Markhabo Shamsiddinova).
