# Supplementary material for: International Survey on Phenylketonuria Newborn Screening
Source: Int J Neonatal Screen. 2025 Feb 26;11(1):18. doi: 10.3390/ijns11010018 (PMC11943362; doi:10.3390/ijns11010018)
Supplement: Supplementary file 1 [file IJNS-11-00018-s001.zip › IJNS-3479530-supplementary.pdf]

Newborn Screeening for Phenylketonuria - International Best Practices

Dear colleague,

We kindly invite you to participate in an international survey to explore the best practices of newborn screening programs for phenylketonuria (PKU) and mild hyperphenylalaninemia.

Despite the fact that PKU screening was first introduced 60 years ago in routine practice and is now almost universally introduced across developed countries, cut-off values for PKU detection (and other newborn screening program characteristics) vary widely between different countries. However, very little insight exists as to why such large differences exist. In addition, most of the PKU guidelines do not specify the optimal PKU screening approach. In many cases, the Phe cut-off values might be too low (e.g., 120 µmol/L), presenting an unnecessary burden for families where children do not require any interventions. There are also examples of countries where cut-off values might not enable the detection of all the children requiring intervention.

In this survey, we aim to obtain further data on how different countries screen for PKU, which would enable the identification and sharing of best practices among countries and form specific recommendations on how to best screen for PKU.

Please take a few minutes to fill out the short questionnaire. It should take you about 15 minutes. If the survey is outside the scope of your expertise, please recommend or directly send it to the most appropriate professional in your country to complete the survey.

If your country has different screening programs in regions/provinces/autonomous regions, please indicate for which region/province/autonomous region in your country you are answering. If you are answering for the whole country, leave that box blank.

This survey is conducted in association with the International Society for Neonatal Screening (ISNS) and European Reference Network for Hereditary Metabolic Disorders (MetabERN).

Thank you for your participation.

Feel free to contact us through e-mail (urh.groselj@kclj.si) if you have any additional questions.

Sincerely,

Urh Groselj, MD, PhD; Domen Trampuž, MSc.

on behalf of the authors, with approval from the ISNS and MetabERN

---

1. Respondent: ______ ______
   We are asking to get more precise response and to invite the respondants to be group co-authors (if interested).

---

2. Role of respondent: __________________________________

3. Institution of respondent: ________________________

4. Country (name): ________________________

5. Country's population (in mil.): ________________________

6. Region/Province covered by your NBS center (name): ________________________

7. Region/Province's population (in mil.): ________________________

8. The number of screening centers in your country: ________________________

9. The number of all newborns in your country in the year 2021: ________________________

10. The estimated number of screened newborns in your country in the year 2021: ________________________

11. The number of all newborns in the region/province covered by your NBS center in the year 2021 ________________________

12. The estimated number of screened newborns in the region/province covered by your NBS center in the year 2021 ________________________

SCREENING AND CUT-OFF VALUES-RELATED QUESTIONS

13. In what year was the PKU screening scheme implemented nationwide in your country?

- ◯ In year ______
- ◯ Information unknown

14. What is the current PKU screening method?

- ◯ Fluorometric
- ◯ Enzymatic
- ◯ MS/MS
- ◯ Radioimmunoassay
- ◯ Bacterial inhibition assay
- ◯ Other: ______

14.1 Optional: Which kit do you use in screening for PKU - what kind? (e.g. derivatized/non-derivatized, in-house method, specific kit name if possible):

____

15. In what year was the current method implemented?

____

16. What is recommended age (in hours) for first sampling for PKU screening?

____

17. What was the initial Phe cut-off value of the currently used method? (please provide units)

____

18. How did you establish the aforementioned Phe cut-off value?

- ◯ From the literature
- ◯ Experimentally
- ◯ Other: ______

19. Do you have a policy of regularly updating cut-off values? (date format DD-MM-YYYY)

- ◯ Yes. We update our cut-off values every ______ years
- ◯ Occasionally, we updated our cut-off values ______
- ◯ No. We still use our initial cut-off value since ______

19.1 Why did you last change your cut-off values?

- ◯ Regular update
- ◯ High false-positive rate
- ◯ Method or reagent kit change swicth ______ ______
- ◯ Other: ______

20. If you differentiate between borderline results and positive results, please define Phe cut-off values: (with units)

- ☐ We do not differentiate. We issue either a positive or a negative result.
- ☐ ______ - ______

20.1. What are the next steps if the result exceeds the cut-off value for a borderline result? Mark all of the answers that apply.

- ☐ Repeat analysis from the initial sample card but from a different DBS
- ☐ Repeat analysis from the initial sample card and the same DBS
- ☐ Ask for a new sample card before repeating the analysis from the initial card
- ☐ Ask for a new sample card
- ☐ Perform confirmatory analysis from a new sample before repeating screening (recall)
- ☐ Perform confirmatory analysis new sample (recall)
- ☐ Perform confirmatory analysis from initial sample before repeating screening (second tier)
- ☐ Perform confirmatory analysis from initial sample (second tier)

21. What are the next steps if the result exceeds the cut-off value for a positive result? Mark all of the answers that apply.	☐ Repeat analysis from the initial sample card but from a different DBS<br>☐ Repeat analysis from the initial sample card and the same DBS<br>☐ Ask for a new sample card before repeating the analysis from the initial card<br>☐ Ask for a new sample card<br>☐ Perform confirmatory analysis from a new sample before repeating screening (recall)<br>☐ Perform confirmatory analysis new sample (recall)<br>☐ Perform confirmatory analysis from initial sample before repeating screening (second tier)<br>☐ Perform confirmatory analysis from initial sample (second tier)
22. Do you test for galactosemia if a newborn has elevated levels of Phe?	◯ Yes<br>◯ Yes in some cases ______<br>◯ No
23. Do you test for BH4 deficiency if a newborn exceeds Phe cut-off value?	◯ Yes in all cases<br>◯ Yes in some cases ______<br>◯ No
24. Do you use Phe/Tyr ratio cut-off value in PKU-screening?	◯ Yes. Our Phe/Tyr ratio cut-off value is ______<br>◯ Yes in some cases ______<br>◯ No.
25. Do you use any other resources in helping with the interpretation (e.g. CLIR)	◯ Yes<br>◯ No
Please specify other resources in helping with the interpretation (e.g. CLIR)	________________________________
26. What confirmatory methods does your center use? Mark all of the answers that apply.	☐ Phe and Tyr from blood plasma<br>☐ Detailed amino acid analysis from blood plasma (amino acid profile)<br>☐ Genetic analysis from DBS<br>☐ Genetic analysis from EDTA blood<br>☐ Dihydrobiopterin reductase activity in red blood cells from DBS<br>☐ Measurement of neopterin and biopterin after tetrahydrobiopterin loading test<br>☐ We outsource the following tests: ______
27. What is your recall rate? If an exact number is not available, provide an estimate. (Recall rate meaning the frequency with which newborns have to be brought back to the hospital for further testing due to a positive PKU screening result. Expressed as the number of recalled newborns per 1000 newborns)	__________________________
28. How do you define a true positive case of PKU	◯ Phe is persistently above cut-off value (for mild hyperphenylalaninemia)<br>◯ Pathologic mutations in both PAH alleles<br>◯ A need for diet therapy<br>◯ Other criteria ______

29. What is the cut-off value for introducing diet therapy in newly diagnosed patients of PKU? ______ ______

30. In case of publication I consent to be listed (with the data I have provided), as one of the group co-authors of the publication (but only in case if that would be applicable):

◯ Yes
◯ No
